# DDX24 Negatively Regulates Cytosolic RNA-Mediated Innate Immune Signaling

**DOI:** 10.1371/journal.ppat.1003721

**Published:** 2013-10-31

**Authors:** Zhe Ma, Robert Moore, Xiangxi Xu, Glen N. Barber

**Affiliations:** 1 Sheila and David Fuente Graduate Program in Cancer Biology, University of Miami Miller School of Medicine, Miami, Florida, United States of America; 2 Department of Cell Biology, University of Miami Miller School of Medicine, Miami, Florida, United States of America; 3 Sylvester Comprehensive Cancer Center, University of Miami Miller School of Medicine, Miami, Florida, United States of America; University of North Carolina at Chapel Hill, United States of America

## Abstract

RIG-I-Like Receptors (RLRs) sense cytosolic viral RNA to transiently activate type I IFN production. Here, we report that a type I IFN inducible DExD/H helicase, DDX24, exerts a negative-regulatory effect on RLR function. Expression of DDX24 specifically suppressed RLR activity, while DDX24 loss, which caused embryonic lethality, augmented cytosolic RNA-mediated innate signaling and facilitated RNA virus replication. DDX24 preferentially bound to RNA rather than DNA species and influenced signaling by associating with adaptor proteins FADD and RIP1. These events preferentially impeded IRF7 activity, an essential transcription factor for type I IFN production. Our data provide a new function for DDX24 and help explain innate immune gene regulation, mechanisms that may additionally provide insight into the causes of inflammatory disease.

## Author Summary

Innate immunity is the first and most rapid host defense against virus infection. Viral RNAs, which are generated during RNA virus replication in host cells, can be recognized through RIG-I-Like Receptors (RLRs) to transiently produce type I interferon, which further induce abundant interferon stimulated genes (ISGs) to clear viral infection. However, uncontrolled innate immune responses cause inflammatory diseases that are detrimental to the host. Therefore, a balanced innate immune response is critical to maintain homeostasis of the host. Thus, RLR signaling is tightly regulated by both positive and negative regulators. DDX24, a helicase reported in this study, is an ISG that exerts an inhibitory effect on RLR dependent signaling. DDX24 hijacked adaptor proteins FADD and RIP1 in host cells to suppress viral RNA dependent interferon production and facilitated RNA virus replication in certain cells. Moreover, DDX24 deficient mouse embryos exerted early embryonic lethality, suggesting an important role for this helicase, perhaps in addition to regulating RLR signaling. In all, our results elucidate the role of DDX24 in RLR dependent signaling, and may shed light on innate immune gene regulation.

## Introduction

The principal purpose of the immediate innate immune response is the rapid synthesis and secretion of type I interferons (IFNβ and IFNα) as well as inducing other key host defense genes [1], [2]. Innate immune signaling is initiated by pattern recognition receptors (PRRs) that specifically recognize pathogen-associated molecular patterns (PAMPs), which are unique to microbes and rarely found in the host [3]. Upon virus infection, virus-associated PAMPs such as genomic DNA or RNA can be recognized by PRRs, which initiate signaling events leading to the synthesis of type I IFN and to the transcription of other IFN-inducible genes (ISG's) in a paracrine or autocrine manner [1], [3]. Three major PRRs participating in the recognition of viral PAMPs have been identified as the Toll-like receptors (TLRs), retinoic acid-inducible gene I-like (RIG-I) receptors (RLRs) and cytosolic DNA receptors [4]–[6]. TLR3, 7, 8 and 9 are TLRs responsible for recognizing viral nucleic acid species. TLR3 is known to recognize dsRNA, while TLR7 and TLR8 recognize ssRNA, and TLR9 senses unmethylated CpG DNA [3]. The RLR family comprises three receptors, RIG-I, melanoma differentiation-associated gene 5 (MDA5) and laboratory of genetics and physiology 2 (LGP2) [5]. RIG-I recognizes 5′-triphosphate RNA and short forms of the synthetic dsRNA analog poly I:C, whereas MDA5 is mainly responsible for recognizing longer dsRNA species [7]–[9]. LGP2 has also been shown to exert anti-viral properties [10], [11]. The mechanisms of cytosolic viral DNA recognition is the least characterized pathway but is known to involve AIM2 which activates inflammatory response [12], [13] and STING (TMEM173/MITA/MPYS) which has been shown to be critical for cytosolic DNA triggered type I IFN production as well as pro-inflammatory gene production [14]–[17]. Furthermore, NLRs (nucleotide-binding domain, leucine-rich repeat containing), key modulators of the inflammasome, also recognize dsRNA. Rather than inducing IFN, they are important for triggering IL-1 beta production, and play important roles in the host response to a wide range of microbial pathogens, inflammatory diseases, and autoimmune disorders [18].

Upon recognition of cytosolic viral RNAs, RLRs are recruited to the adaptor protein IPS-1 (MAVS, Cardif, VISA) located on mitochondria and peroxisomes, through the mitochondrial-associated membrane (MAM), a distinct membrane compartment that links the endoplasmic reticulum to mitochondria [19]–[24]. IPS-1 subsequently recruits TBK-1/IKKi, which phosphorylates IFN-regulatory factor 3 (IRF3) and 7 (IRF7). Phosphorylated IRF3 and IRF7 dimerize and translocate into the nucleus to trigger the production of type I IFN and other primary innate immune genes. NF-κB and AP-1 are also activated in this pathway and required for the optimal type I interferon production [25], [26].

RLR signaling is facilitated by co-regulators such as Fas associated death domain (FADD) and receptor interacting protein 1 (RIP1), originally identified as crucial players in apoptotic and inflammatory signaling pathways [27], [28]. It has been demonstrated that RIP1 and FADD can form a complex with TRADD and IPS-1 following RNA virus infection to co-ordinate signaling [29]. Indeed, loss of FADD or RIP1 leads to defective type I IFN production and significantly increased susceptibility to RNA virus infection [28], [30]. RIP1 may exert its effects with FADD by associating with IRF7 to promote its activation. IRF7 activity is known to be reduced in RIP1 deficient MEFs, again suggesting that RIP1 is a positive regulator in RLR dependent signaling [31]. However, the detailed mechanisms of RLR signaling that involves FADD and RIP1 remains to be fully clarified, including the mechanisms of negative regulation.

Here, we report that an IFN-inducible helicase referred to as DDX24 is able to negatively regulate the RLR-signaling pathway. DDX24 attenuates the RLR signaling possibly through competing with RIG-I for binding of RNA. Moreover, it also disrupts the IRF7/RIP1 interaction, which is required for robust innate immune gene activation. Our data provides significant molecular insight into the control of innate immunity and may provide information into the causes of inflammatory disease.

## Results

### Association of IFN-inducible DDX24 with FADD

**Figure 1:**
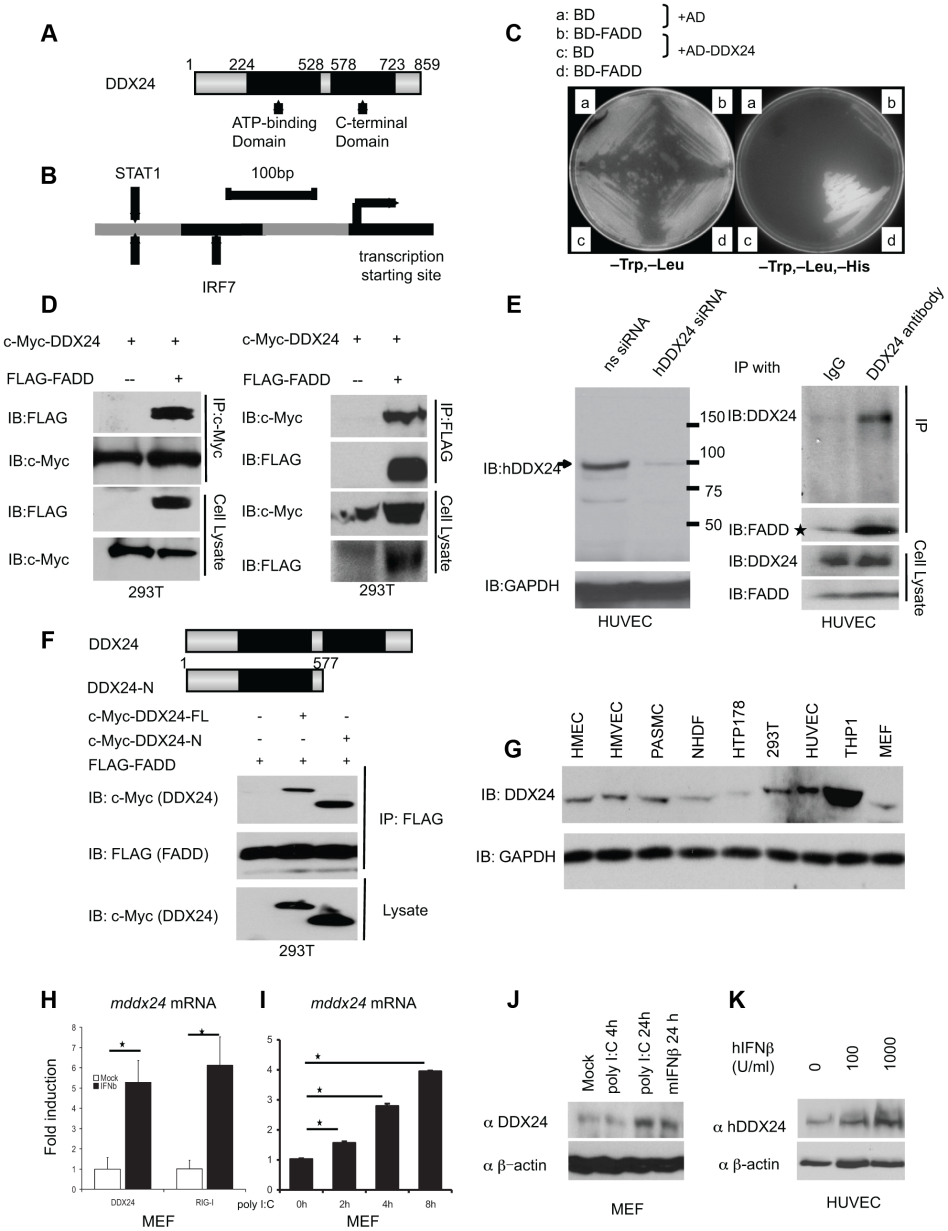
DDX24 is a FADD-associating interferon inducible helicase. (A) Schematic of human DDX24 (hDDX24) indicating helicase domains. (B) Schematic of human DDX24 promoter region, indicating STAT1 and IRF7 binding site. (C) Yeast two hybrid assay confirming FADD and DDX24 interactions. (D)293T cells were transiently transfected with c-Myc-DDX24 and FLAG–FADD or control plasmid. Lysates were immunoprecipitated (IP) and immunoblotted (IB) using antibodies to c-Myc or FLAG. (E) On the left, Immunoblot analysis of DDX24 in HUVEC cells treated with DDX24 siRNA or control ns (non-specific) siRNA. On the right, endogenous human DDX24 associates with FADD in HUVEC. Lysates of HUVEC cells were immunoprecipitated with anti-FADD or mouse IgG serum. The immunoprecipitates were analyzed by immunoblot with anti-DDX24 or anti-FADD (top). The expression levels of the endogenous DDX24 and FADD were detected by immunoblot analysis (bottom). (F) Schematic of human DDX24 (hDDX24) indicating helicase domains. 293T cells were transiently transfected with c-Myc-DDX24-FL, c-Myc-DDX24-N or control plasmid with FLAG-FADD, Lysates were immunoprecipitated (IP) using antibodies to FLAG and immunoblotted (IB) using antibodies to c-Myc. (G) Immunoblot analysis of DDX24 in varies human cells or cell lines and MEFs, normalized byGAPDH. (H) MEFs were left untreated or treated with mIFNβat 100 U/ml. Mouse *ddx24* and *rig-i* mRNA were analyzed by RT-PCR. (I) MEFs were left untreated or treated with poly I:C for the indicated time. Mouse *ddx24* mRNA was analyzed by RT-PCR. (J) MEFs were left untreated or treated with poly I:C, mIFNβ for the indicated time. Mouse DDX24 and β-actin were detected by immunoblot analysis. (K) HUVEC cells were left untreated or treated with hIFNβ at increasing does. Human DDX24 and β-actin were detected by immunoblot analysis. Data from (H)(I) are presented as means±s.e. from three independent experiments. * indicates P<0.05. ** indicates P<0.01.

Given that FADD is important in regulating innate immune signaling, we searched for proteins that may mediate FADD activity. We noted that FADD had been reported by proteomic analysis to be potentially associated with a helicase, referred to as DDX24 [32], [33]. DDX24, an 859 amino acid Asp-Glu-Ala-Asp (DEAD)-box ATP-dependent RNA helicase, lacks CARD domains typical of RIG-I and MDA5 though has an N-terminal region rich in glutamic acid and lysine residues (Figure 1A and Figure S1A). Over 90 percent of homology at the amino acid level is shared between human DDX24 and mouse DDX24 (Figure S1A). It is also of interest to note that unlike the majority of DEAD-helicases, DDX24 has several potential interferon-regulated transcription sites in its promoter region, similar to RIG-I, MDA5 and LGP2, such as STAT1 and IRF7 binding sites (Figure 1B). To further evaluate the possible association of DDX24 with FADD, we used FADD as bait in a yeast-two hybrid assay, with DDX24 as prey. This study confirmed that full length DDX24 could specifically associate with FADD in this system (Figure 1C). To complement this approach, we carried out co-immunoprecipitation analysis by co-overexpressing DDX24 and FADD in 293T cells and found DDX24 and FADD could interact with each other (Figure 1D). We next examined whether endogenous FADD and DDX24 could co-immunoprecipitate from primary human HUVEC (human umbilical vein endothelial) cells. First, we confirmed that the available antibody could recognize DDX24 in HUVECs. To accomplish this, we suppressed DDX24 expression in HUVECs by RNAi and then carried out an immunoblot analysis. We observed a single band of molecular weight of approximately 100 kDa in untreated cells that was not evident in the RNAi treated HUVECs, confirming DDX24 expression in this cell-type (Figure 1E, left panel). Subsequent analysis using anti-DDX24 antibody similarly indicated co-association of DDX24 and FADD (Figure 1E, right panel). An extended analysis suggested that FADD associated with the amino terminal region of DDX24 containing the DExD/H box helicase ATP binding domain (Figure 1F). Expression studies suggested that DDX24 was expressed in a wide variety of cells (Figure 1G and Figure S1B). We observed that DDX24 and FADD were localized both nucleus and cytoplasm by immunofluorescent and fractionation experiments (Figure S2A and S2C). Interestingly, we observed elevated cytoplasmic DDX24 6 and 9 hours following poly I:C treatment (Figure S2B). Consistently, increased levels of DDX24 co-precipitated with FADD after 6 hours of poly I:C treatment in MEF, suggesting a stronger DDX24/FADD association following RLR activation (Figure S2D).Treatment of murine embryonic fibroblasts (MEFs) or HUVECs with type I IFN or poly I:C confirmed that DDX24 was IFN-inducible, similar to RIG-I, and the induction was STAT1 dependent as predicted (Figure 1H–K and Figure S2E). Thus, DDX24 appears to be an interferon-inducible DEAD-box helicase that can associate with FADD.

### DDX24 impedes RLR-signaling

**Figure 2:**
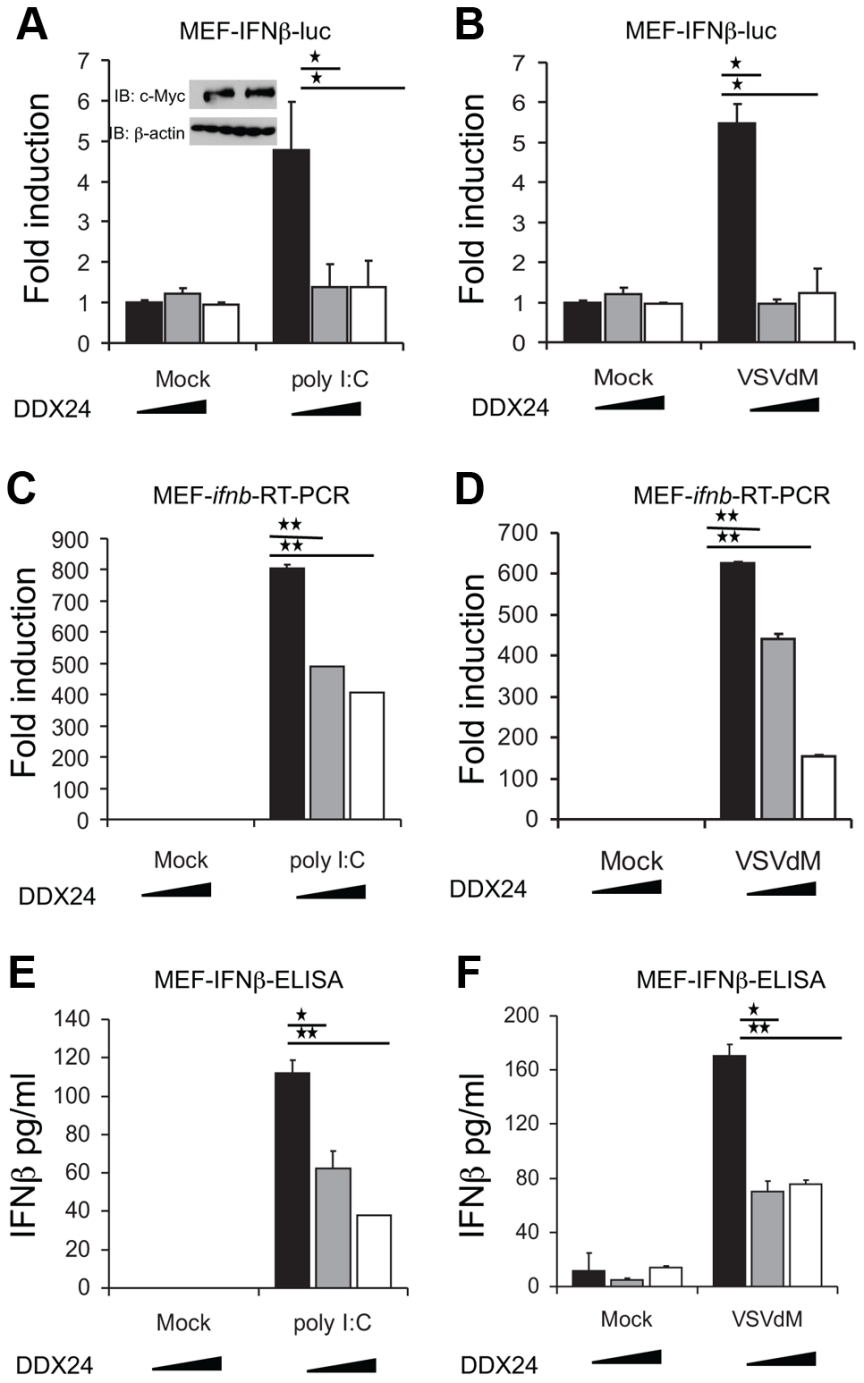
DDX24 inhibits dsRNA induced RLR signaling. (A) DDX24 inhibits poly I:C-induced activation of the IFNβ promoter in a dose-dependent manner in MEFs. MEF cells transfected with vector or c-Myc-DDX24 (200 µg or 400 µg) were transfected with 2 µg/ml poly I:C overnight before testing for luciferase expression. Expression of c-Myc tagged DDX24 in MEFs was monitored using anti c-Myc antibody, normalized by β-actin. (B) DDX24 inhibits VSVdM-induced activation of the IFNβ promoter in a dose-dependent manner in MEFs. MEF cells transfected with vector or c-Myc-DDX24 (200 µg or 400 µg) were infected by VSVdM at MOI 10 overnight before testing for luciferase expression. (C) DDX24 inhibits poly I:C-induced endogenous *ifnb* transcription in MEFs. MEF cells transfected with vector or c-Myc-DDX24 (200 µg or 400 µg) were transfected with 2 µg/ml poly I:C for 6 hours before testing *ifnb* RNA by RT-PCR. (D) DDX24 inhibits VSVdM-induced endogenous *ifnb* transcription in MEFs. MEF cells transfected with vector or c-Myc-DDX24 (200 µg or 400 µg) were infected by VSVdM at MOI 10 for 6 hours before testing *ifnb* RNA by RT-PCR. (E) DDX24 inhibits poly I:C-induced endogenous IFNβ protein expression inMEFs. MEFs were transfected with control plasmid or DDX24 expressing plasmid. Twenty four hours after transfection, cells were transfected with 2 µg/ml poly I:C or left untreated overnight. Endogenous IFNβ were analyzed by ELISA. (F) DDX24 inhibits VSVdM-induced endogenous IFNβ protein expression inMEFs. MEFs were transfected with control plasmid or DDX24 expressing plasmid. Twenty four hours after transfection, cells were infected with VSVdM at MOI = 1 or left uninfected overnight. Endogenous IFNβ were analyzed by ELISA. Data are presented as means±s.e. from three independent experiments. * indicates P<0.05. ** indicates P<0.01.

To evaluate the importance of DDX24 in possible innate immune signaling regulation, we overexpressed DDX24 in MEF cells and confirmed that the type I IFN inducer, poly I:C, could initiate the transcription of an IFNβ promoter driven luciferase construct (Figure 2A). However, we noted that the overexpression of DDX24 could inhibit poly I:C's ability to activate the IFNβ promoter (Figure 2A). Similarly, DDX24 was seen to inhibit the ability of vesicular stomatitis virus (VSVdM, with a defective matrix protein that enables virus-mediated type I IFN production) to activate IFNβ-luciferase production [34] (Figure 2B). This was confirmed at the level of IFNβ mRNA expression and endogenous protein expression (Figure 2C–2F). Thus, DDX24 may negatively regulate innate immune signaling processes that activate the type I IFN promoter.

**Figure 3:**
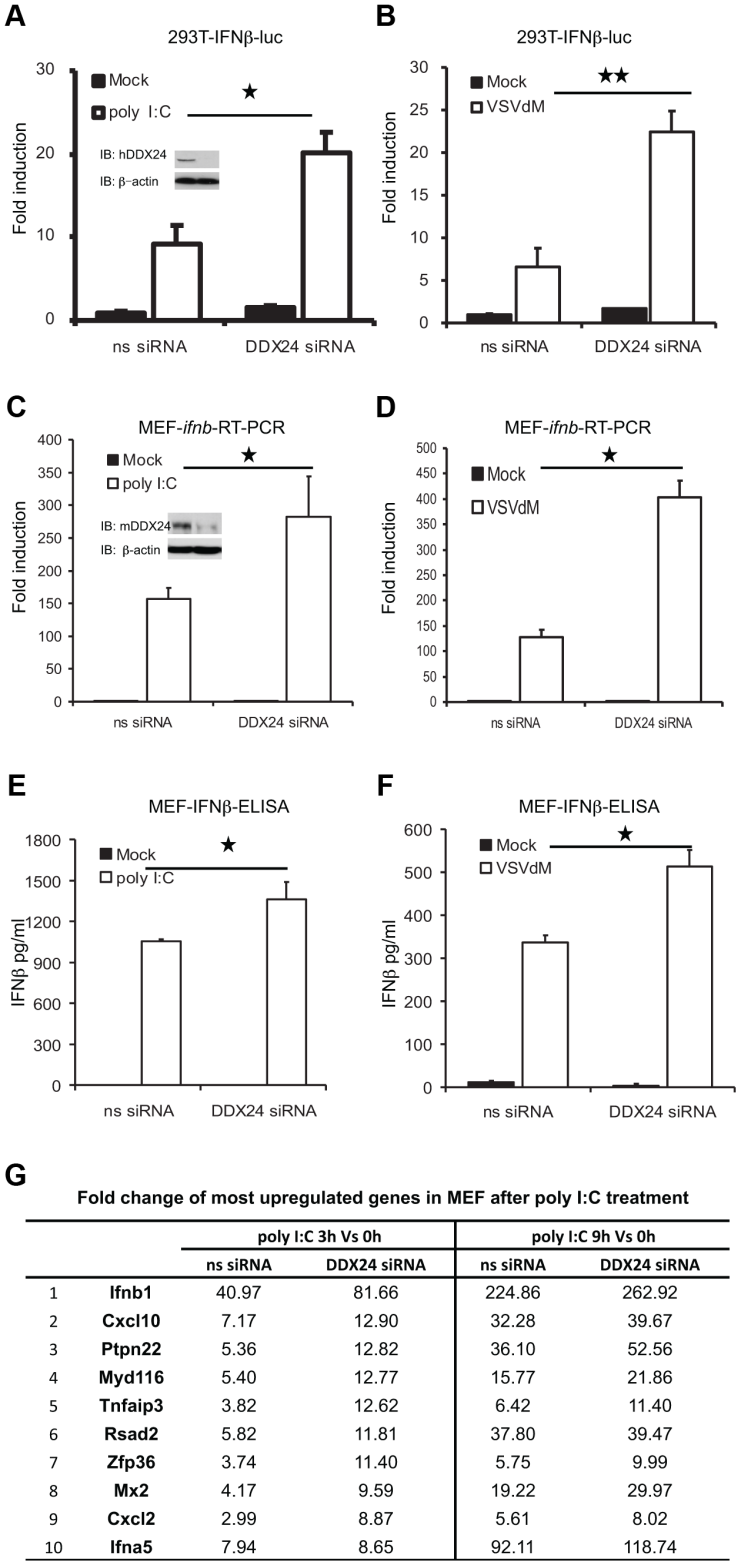
SiRNA-mediated knockdown of DDX24 enhances dsRNA induced RLR signaling. (A) Effects of DDX24 RNAi on poly I:C-induced activation of the IFNβ promoter in 293T cells. 293T cells were transfected with ns or human *ddx24* siRNA. Forty eight hours after transfection, cells were left untreated or transfected with poly I:C overnight before luciferase assays were performed. 293T cell lysates were analyzed by immunoblotting with the indicated antibodies to ensure the knockdown of hDDX24. (B) Effects of DDX24 RNAi on VSVdM-induced activation of the IFNβ promoter in 293T cells. 293T cells were transfected with ns or human *ddx24* siRNA. Forty eight hours after transfection, cells were left untreated or infected with VSVdM overnight before luciferase assays were performed. (C) Effects of DDX24 RNAi on 6 hours treatment of poly I:C-induced endogenous *ifnb* transcription in MEFs by RT-PCR. MEF cell lysates were analyzed by immunoblotting with the indicated antibodies to ensure the knockdown of mDDX24. (D) Effects of DDX24 RNAi on 6 hours infection of VSVdM-induced endogenous *ifnb* transcription in MEFs by RT-PCR. (E) Effects of DDX24 RNAi on overnight treatment of poly I:C-induced endogenous IFNβ production in MEFs by ELISA. (F) Effects of DDX24 RNAi on overnight infection of VSVdM-induced endogenous IFNβ production in MEFs by ELISA. (G) Gene array indicating most up-regulated genes in MEF treated with poly I:C at 3 hours and 9 hours VS non treatment. Data from (A)(B)(C)(D)(E)(F) are presented as means±s.e. from three independent experiments. * indicates P<0.05. ** indicates P<0.01.

To further evaluate this possibility, we treated 293T cells with RNAi that targeted DDX24. After confirming knockdown by immunoblot, we observed that loss of DDX24 led to an increase in polyI:C's ability to activate luciferase under control of the IFNβ promoter (Figure 3A). A similar effect was observed following infection with VSVdM (Figure 3B). We complemented this approach by knocking down DDX24 in MEF cells and observed a comparative effect (Figure 3C–3F). These data again indicate that DDX24 can exert a negative-regulatory effect on cytosolic RNA signaling events in the cell. Microarray analysis of polyI:C transfected MEF cells treated with or without anti-DDX24 RNAi confirmed that DDX24 suppressed IFNβ production as well as IFN-inducible genes such as CXCL10 (Figure 3G). Thus, DDX24 may be a negative regulator of RNA-mediated type I IFN transcriptional activation (Figure 3G).

**Figure 4:**
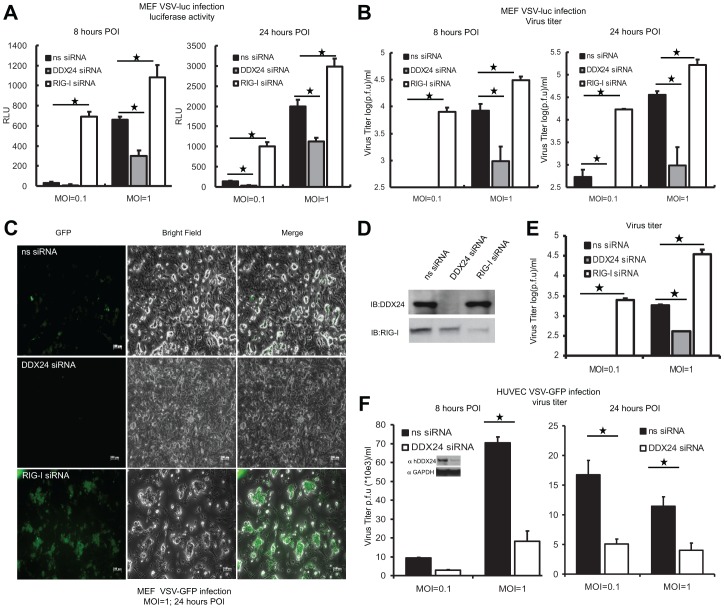
SiRNA-mediated knockdown of DDX24 inhibits VSV replication. (A) Loss of mDDX24 affects VSV-luc replication in MEFs. MEFs transfected with ns, mDDX24 or RIG-I siRNA for 72 hours were infected by VSV-luc at M.O.I. = 0.1 or 1. Eight hours and 24 hours post infection, luciferase activities from infected MEF cell lysate were detected. (B) Virus titer from supernatant of (A). (C) Loss of mDDX24 affects VSV-GFP replication in MEFs. Fluorescence microscopy (GFP) of ns, mDDX24 or mRIG-I siRNA treated MEFs following with VSV-GFP infection 24 hours post infection at M.O.I 1. (D) Knockdown efficiency check in MEF. DDX24 and RIG-I antibody are used to detect endogenous DDX24 or RIG-I. (E) Virus titer from supernatant of (C). (F) Loss of hDDX24 affects VSV replication in HUVECs. HUVECs transfected with ns or hDDX24 siRNA for 72 hours were infected by VSV-luc at M.O.I. = 0.1 or 1. Eight hours and 24 hours post infection, plaque assays were performed using supernatants from infected MEFs. HUVEC cell lysates were analyzed by immunoblotting with the indicated antibodies to ensure the knockdown of hDDX24. Data from (A)(B)(E)(F) are presented as means±s.e. from three independent experiments. * indicates P<0.05. ** indicates P<0.01.

Since we observed that loss of DDX24 may augment RNA-mediated type I IFN production, we surmised that DDX24 suppression may in turn repress RNA virus replication because of prevalently high type I IFN levels. Two types of recombinant VSV were used for these studies to help facilitate this analysis. First we suppressed DDX24 in MEFs cells and infected with VSV expressing a luciferase gene (VSV-Luc). We noted that suppression of DDX24 robustly prevented the replication of this virus as determined by luciferase levels and viral titers 8 or 24 hours post infection (Figure 4A and 4B). As a control, we knocked down RIG-I, which is a key sensor of VSV-mediated type I IFN production [5]. Suppression of RIG-I unsurprisingly lead to an increase in VSV-Luc replication opposite to DDX24 suppression (Figure 4A and 4B). Similar results were obtained using VSV expressing GFP (VSV-GFP) in MEFs treated with DDX24 siRNA (Figure 4C–4E). HUVEC cells treated with RNAi targeting DDX24 likewise lead to a decrease in VSV replication (Figure 4F). Collectively, our data indicate that DDX24 exerted suppressive effects on RNA-mediated innate immune signaling events in the cell controlled by RLRs.

### DDX24 can sequester RLR activator RNA

**Figure 5:**
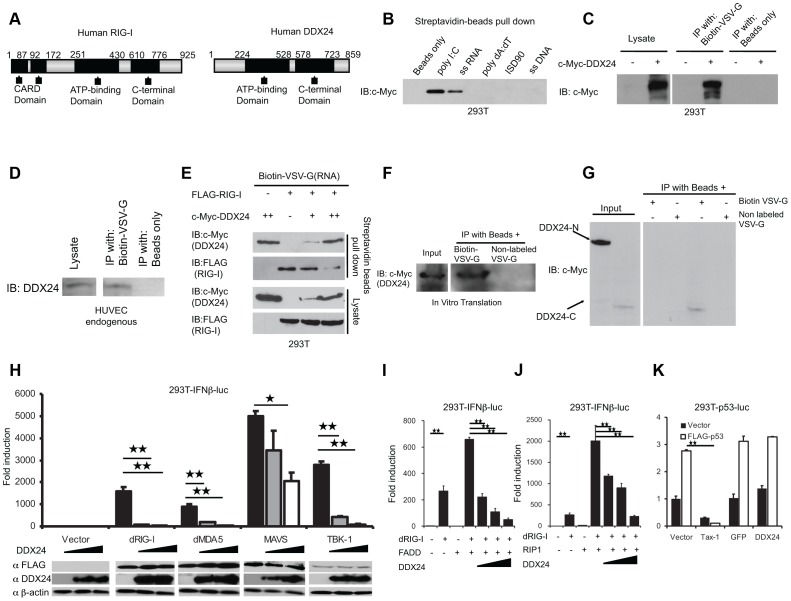
DDX24 can sequester RLR activator RNA. (A) Schematic of human RIG-I and DDX24. (B) DDX24's binding specificity. 293T cells were transfected with c-Myc-tagged DDX24 for 24 hours before lysed. Pull-down assays were performed by incubating 293T lysate with various biotin-conjugated polynucleotides, and then precipitated with streptavidin beads. Bound proteins were analyzed by immunoblotting with anti-c-Myc antibody. (C) ssRNA transcribed from VSV-G cDNA were conjugated with biotin and utilized in pull-down assays similar to (B). (D) Endogenous DDX24 binds to VSV-G RNA. Pull-down assays were performed by incubating HUVEC cells lysate with biotion-VSV-G. Endogenous DDX24 were analyzed by immunoblotting with anti-DDX24 antibody. (E) DDX24 attenuates RIG-I's RNA binding activity. 293T cells were transfected with c-Myc-tagged DDX24 or FLAG-RIG-I as indicated for 24 hours before lysed. Pull-down assay were performed using biotin-VSV-G and bound proteins were applied to immunoblotting. (F) In vitro translated DDX24 binds to VSV-G RNA. Pull-down assays were performed by incubating in vitro translated DDX24 with biotion-VSV-G or non-labeled VSV-G. DDX24 were analyzed by immunoblotting with anti-c-Myc antibody. (G) In vitro translated DDX24 helicase C domain binds to VSV-G RNA. Pull-down assays were performed by incubating in vitro translated c-Myc-DDX24-N or c-Myc-DDX24-C with biotion-VSV-G or non-labeled VSV-G. DDX24mutants were analyzed by immunoblotting with anti-c-Myc antibody. (H) DDX24 inhibits dRIG-I-, dMDA5, IPS-1 and TBK-1 mediated IFNβ promoter activation. (I)(J) DDX24 blocks synergistical effect of FADD/RIP1 with RIG-I. 293T cells were transfected with reporter plasmid and variant plasmids as indicated. Activations of IFNβ promoter were detected 36 hours post transfection. (K) DDX24 does not affect p53 activation. 293T cells were transfected with reporter plasmid and variant plasmids as indicated. Activations of p53 promoter were detected 36 hours post transfection. Data from (H)(I)(J)(K) are presented as means±s.e. from three independent experiments. * indicates P<0.05. ** indicates P<0.01.

RIG-I and MDA5 have been reported to sense viral negative and positive stranded RNA species, respectively, to initiate the activation of NF-κB and IRF3 signaling pathways that results in the production of type I IFN and other primary innate immune genes [9]. Briefly, viral RNA interacts with the helicase domains of RIG-I/MDA5, inducing conformational changes that enable the CARD domains to interact and activate IPS1/MAVS [19]–[22] (Figure 5A). It was thus plausible that DDX24 could suppress RIG-I/MDA5 activity by competing with RNA activators. To evaluate this, we first examined the ability of DDX24 to directly associate with RNA species. Our analysis indicated that c-Myc-tagged DDX24 could be precipitated from 293T cells using biotinylated polyI:C or ssRNA compared to DNA species (Figure 5B–5D and Figure S2). Furthermore, in vitro binding experiments indicate that DDX24 could bind to the VSV-G gene transcript through its helicase C domain (Figure 5F and G). We also observed that DDX24 was able to compete with RIG-I for RNA representing the VSV-G gene transcript (Figure 5E). Thus, it is plausible that DDX24 may exert some negative-regulatory effect by directly competing for RIG-I/MDA5's activators. However, to evaluate whether DDX24 could directly inhibit RIG-I and/or MDA5 by means other than by plausible sequestration of RNA ligands, we transfected dRIG-I or dMDA5 constructs into 293T cells co-transfected with increasing amounts of DDX24. The dRIG-I/dMDA5 constructs we used represented the amino terminal CARD domains (dRIG-I aa 1–284; dMDA5 aa 1–349) that following expression, oligomerize without the requirement of RNA to activate IPS-1/MAVS and type I IFN signaling [5], [30]. Thus, we were also able to determine whether DDX24 impeded RIG-I function independent of RNA. This experiment indicated that DDX24 could indeed inhibit dRIG-I and dMDA5-mediated signaling which is independent of RNA sequestration (Figure 5H). Overexpressed IPS1/MAVS is also known to oligomerize to activate type I IFN activity without the requirement of RIG-I/MDA5. Since similar experimentation indicated that DDX24 could also inhibit IPS1/MAVS signaling, we conclude that DDX24's regulatory influence was also exhibited downstream of RIG-I/MDA5 and IPS1/MAVS interaction (Figure 5H). Indeed, we found that DDX24 could affect TBK1 activity suggesting that this helicase exerts its influence at the levels of RIG-I/MDA5's ability to regulate IRF3/7 dependent type I interferon production.

We had also previously reported that FADD and RIP1 regulate RNA-mediated innate immune signaling [28]. We noted that expression of FADD or RIP1 could greatly facilitate dRIG-I's ability to augment type I IFN production (Figure 5I and 5J). However, DDX24 was again able to inhibit this signaling process (Figure 5I and 5J). This would suggest that DDX24 competes in innate immune complexes comprising FADD/RIP1 that are required for efficient type I IFN signaling. It should be noted that we observed that DDX24 predominantly inhibited type I IFN signaling compared to other signaling pathways. For example, DDX24 did not affect p53 signaling, general *luc* gene transcription or induce cell death (Figure 5K and Figure S4). Thus DDX24 is able to sequester RNA activators of type I IFN activation as well as additionally interfere with downstream signaling events that control RIG-I/MDA5 function.

### DDX24 disrupts RLR's activation of IFN-dependent transcription factor IRF7

**Figure 6:**
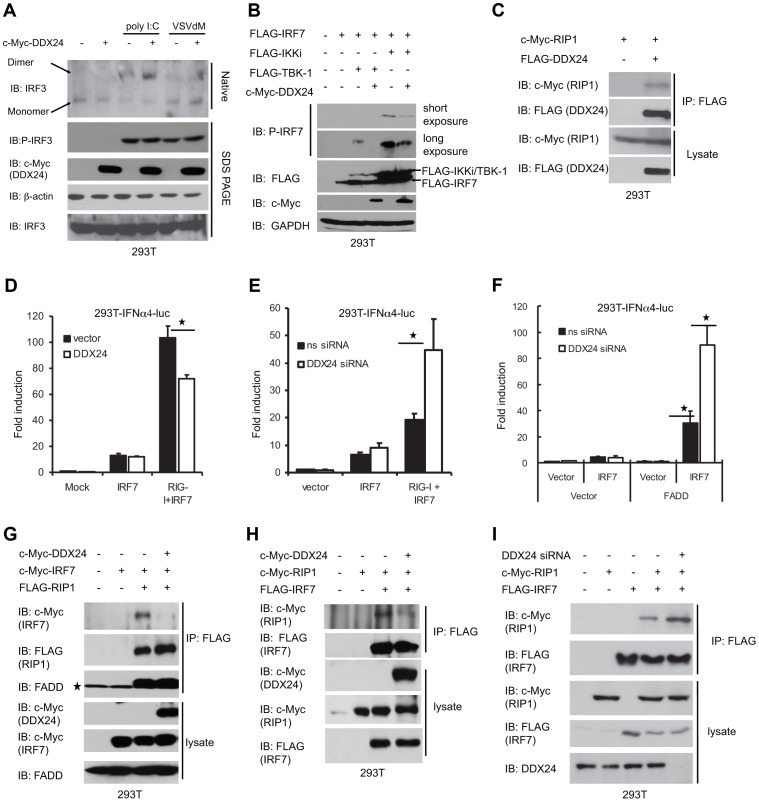
DDX24 interacts with RIP1 and disrupts RLR's activation of IFN-dependent transcription factor IRF7. (A) DDX24's effect on IRF3 phosphorylation. 293T cells transfected with vector or c-Myc-DDX24 were treated with 2 µg/ml poly I:C or infected by VSVdM at MOI 10 for 3 hours. Cell lysates were then prepared, and the dimerization and phosphorylation of IRF3 was analyzed by native or SDS PAGE. (B) DDX24's effect on IRF7 phosphorylation. Immunoblot for detecting IRF7 phosphorylation using 293T cells transfected with c-Myc-tagged DDX24, FLAG-tagged IRF7, TBK-1 and IKKi as indicated. (C) DDX24 interacts with RIP1. 293T cells were transfected with either c-Myc-tagged RIP1 or FLAG-DDX24 as indicated. After 24 h, cells were harvested, and lysates were subjected to co-IP and immunoblotting (IB) with the indicated antibodies. (D) DDX24's effect on RIG-I dependent IFNα4 promoter activation. 293T cells were transfected with IFNα4-luc reporter plasmid and variant plasmids as indicated. Activations of IFNα4 promoter were detected 36 hours post transfection. (E)(F) Loss of DDX24's effect on RIG-I dependent IFNα4 promoter activation. 293T cells were transfected with ns or DDX24 siRNA. Forty eight hours post transfection, cells were transfected with IFNα4-luc reporter plasmid and variant plasmids as indicated. Activations of IFNα4 promoter were detected 36 hours post transfection. (G)(H) DDX24 disrupts RIP1-IRF7 interaction. 293T cells were transiently transfected with variant plasmids as indicated and proper control plasmids. Thirty six hours post transfection, cell lysates were immunoprecipitated (IP) and immunoblotted (IB) using antibodies to c-Myc, FLAG or FADD. (I) Loss of DDX24 enhances RIP1-IRF7 interaction. 293T cells were transiently transfected with ns siRNA or DDX24 siRNA. Forty eight hours later, cells were transfected with variant plasmids as indicated and proper control plasmids. Twenty four hours post transfection, cell lysates were immunoprecipitated (IP) and immunoblotted (IB) using antibodies to c-Myc, FLAG or DDX24.Data from (D)(E)(F) are presented as means±s.e. from three independent experiments. * indicates P<0.05. ** indicates P<0.01.

The regulation of IRF3 and NF-κB pathways are complex though are known to involve RIG-I/MDA5 invoking TBK1 to principally phosphorylate IRF3 which then along with activation of the NF-κB pathway activates IFNβ transcription [3]. These events produce type I IFN-inducible IRF7 that binds to and activates several IFNα genes to augment type I IFN production in a positive feedback manner [35]. To additionally evaluate the mechanisms of DDX24 activity, we inquired whether DDX24 affected IRF3 or IRF7 activity. However, we observed that DDX24 did not affect poly I:C or VSVdM dependent IRF3 phosphorylation or dimerization (Figure 6A). Furthermore, we did not observe an inhibition of constitutively activated IRF3(SA)–mediated induction of IFNβ-luc during overexpression of DDX24 (Figure S5B and Figure S5C). To next evaluate the influence of DDX24 on IRF7 function, we examined the effects of DDX24 on an IFNα4 promoter driving luciferase which is strongly activated by IRF7 rather than IRF3 [36]. This experiment indicated that overexpression of DDX24 in 293T cells inhibited the ability of RIG-I and IRF7 to fully activate the IFNα4 promoter (Figure 6D). Conversely, loss of DDX24 in 293T cells by RNAi treatment enhanced RIG-I and IRF7's ability to activate the IFNα4 promoter (Figure 6E). Overexpression of FADD was observed to facilitate IRF7 signaling, which was significantly increased in the absence of DDX24 (Figure 6F). To extend this analysis, we evaluated whether DDX24 could affect TBK1/IKKi's ability to phosphorylate IRF7. Co-expression analysis confirmed that DDX24 could affect IRF7 phosphorylation (Figure 6B). Thus, DDX24 could influence IRF7 function, which is a pivotal positive regulator of type I IFN production.

Given this we next examined whether DDX24 disrupted FADD or RIP interactions with downstream components of the RLR pathway, which would eventually lead to attenuated IRF7 activation. We first tested the interaction of FADD and IRF7, but no association was observed by co-immunoprecipitation after overexpressing both proteins in 293T cells (data not shown). However, it has been reported that RIP1 could recruit IRF7 to the signaling complexes to positively regulate IRF7 activation [31]. We thus explored the association of DDX24, RIP1 and IRF7 through co-immunoprecipitation experiments. Indeed, c-Myc-tagged DDX24 was observed to interact with RIP1 (Figure 6C). However, no interactions between DDX24 and IRF7 were detected (data not shown). Since IRF7 and DDX24 have been reported to bind to RIP1, it is possible that DDX24 could disrupt the interactions between IRF7 and the RIP1 kinase. To examine this, we co-expressed RIP1 and IRF7 with or without DDX24. This experiment confirmed that RIP1 could associate with IRF7 and that this event could be disrupted in the presence of DDX24 (Figure 6G). It is therefore plausible that DDX24 may compete with RIP1 to bind IRF7. Reciprocal co-IP experiments confirmed that RIP1 precipitated with IRF7, an event that was impeded in the presence of DDX24 (Figure 6H). Consistent with our findings, we observed increased RIP1 interaction with IRF7 when we knocked down DDX24 by siRNA in 293T cells (Figure 6I). Collectively our data indicates that DDX24 negatively regulates RLR signaling at least in part by affecting RIP1/IRF7 interactions.

### DDX24 loss causes embryonic lethality

To study the function of DDX24 in vivo, DDX24 knockout mice were generated using a gene trapping strategy. Mouse embryonic stem (ES) cells with the DDX24 genomic locus disrupted by a β-galactosidase/neomycin cassette placed between exons 6 and 7 were microinjected into C57BL/6 blastocysts to create chimeric mice (Figure S4A). Although DDX24^+/−^ mice appeared normal and were fertile, no viable homozygous mutant mice were observed in the first 300 pups derived from DDX24^+/−^ intercrosses, suggesting that DDX24 deficiency results in embryonic lethality (Table 1 and Figure S4B). In fact, we were not able to retrieve DDX24^−/−^ MEFs from over 200 embryos isolated between E12.5 to E9.5 (Table 1 and Figure S4C). We were only able to identify DDX24^−/−^ embryos between E3.5 and E8.5 suggesting an embryonic lethal occurrence between these times (Figure S4D). By histological analysis, we were able to identify embryos with severe embryogenesis defect at E7.5 (Figure 4E). These data suggest that loss of DDX24 is lethal to embryos before E8.5, suggesting an important role for this helicase, perhaps in addition to facilitating RLR signaling.

**Table 1:** Genotypes of embryos/mice derived from DDX24+/− intercrosses.

*Stage*	*+/+*	*+/−*	*−/−*	*Resorptions*	Total
E9.5	14	27	0	12	53
E10.5	14	25	0	7	46
E11.5	11	23	0	2	36
E12.5	8	16	0	1	25
Postnatal (4 weeks)	112	189	0	N/A	301

## Discussion

It has been previously reported that RIG-I/MDA5 associates with FADD, RIP1 and TRADD in a signaling complex that also involves IPS-1 and which is required for the stimulation of host defense genes [1], [29]. Our data here indicate that an uncharacterized helicase DDX24 may negatively regulate these processes. DDX24 belongs to the DExH/D family, which contains at least 59 proteins conserved from bacteria to humans. DExH/D helicases are broadly involved in many RNA related processes such as transcription, translation, ribosome biogenesis and RNA transportation [37]. In addition, RIG-I, MDA5 and LGP2 have been reported to be key sensors in RNA-virus mediated innate immune signaling processes. However, several other RNA helicases have also been implicated in the regulation of host defense processes such as DHX9, DDX60 and DDX3x which reportedly act as alternate RNA sensors in myeloid dendritic (mDCs) among other cells [38]–[40]. DDX1/DDX21/DHX36 complex may similarly be involved with sensing both short and long poly I:C via the adaptor TRIF [41]. Further, DHX9 and DHX36 have been reported to sense cytosolic CpG-DNA via MyD88 in pDCs [42]. Finally, DDX41 has been reported to sense intracellular DNA [43].

Although a majority of these helicases are able to bind RNA or DNA and may act as sensors, it is also possible that certain members such as DDX3x may also function downstream of nucleic acid recognition to affect multi-protein signaling complexes required for efficient primary innate immune gene transcription [44], [45]. Although we observed that DDX24 could specifically bind to dsRNA or ssRNA, DDX24 did not show a binding affinity to DNA species, which suggests a preferable role in RNA dependent signaling. Furthermore, although DDX24 exerts RNA binding activity that is similar to the RLR's, DDX24 was able to inhibit the function of constitutively active RLR's that lacked RNA binding ability, such as dRIG-I and dMDA5. This data would suggest that aside from being able to sequester RNA activators, DDX24 may additionally act as a competitive protein to disrupt the appropriate formation of downstream signaling complexes. For example, it has been reported that RIP1 is able to associate with IRF7 and is important for IRF7 activation. Possibly, RIP1 helps to recruit IRF7 to signaling complexes comprising TBK-1/IKKi. In this study, we have confirmed an interaction between RIP1 and IRF7 and demonstrated specific inhibition of this interaction by DDX24. Cells overexpressing DDX24 exhibited attenuated TBK-1/IKKi dependent IRF7 phosphorylation, which would be in agreement with this model. Additionally, we investigated the potential interacting partner of DDX24, but we did not observe direct evidence of DDX24 binding to IPS-1 or TRADD (data not shown, Figure S2F), which are reported as FADD binding molecules [19], [29].

Although no direct interactions between FADD and IRF7 were observed, FADD was able to greatly facilitate IRF7-dependent IFNα4 promoter activation. Moreover, this regulatory function is significantly enhanced in the absent of DDX24, indicating the requirement for FADD on IRF7 activity. Given the fact that RIG-I associates with both FADD and RIP1 without disrupting the interaction, it is plausible to propose that FADD-RIP1 association acts as scaffold that facilitates the recruitment of IRF7 to the IPS-1 based signaling complex. Losing either FADD or RIP1 may lead to a disruption of the signaling the complex, which could potentially lead to insufficient recruitment of IRF7 to this complex. DDX24, anchored into this complex by interacting with FADD and RIP1 likely disrupts the binding of IRF7, and thus negatively regulates this pathway.

DDX24 appears to be relatively ubiquitously expressed, not just prevalent in hematopoietic lineages, and it can be induced by IFNβ and poly I:C treatment. DDX24 was strongly associated with the nucleolus. However, our results from immunofluorescent and fractionation experiments suggest that FADD and DDX24 converge in the cytoplasm in quantities sufficient enough to regulate innate immune signaling events. Interestingly, this DDX24/FADD association is strengthened after the cells were treated with poly I:C for 6 hours as detected by co-immunoprecipitation in MEF cells. Consistently, we have also observed increased DDX24 in the cytoplasm in our immunofluorescent experiments. This suggests the translocation of DDX24 following RLR activation or that the cytoplasmic DDX24 is induced by poly I:C treatment. Similar to the immunofluorescent experiments, we observed elevated levels of cytoplasmic DDX24 after 24 hours of poly I:C stimulation. However, we did not observe significant changes in levels of nuclear DDX24, which suggests a function unrelated to IFN signaling for nuclear DDX24 that remains unclear.

Moreover, the interferon inducible characteristic of DDX24 provides extra clues on the mechanism of DDX24's regulation of RLR signaling. Indeed, we observed induction of poly I:C-inducible genes following 9 hours poly I:C treatment when DDX24 was knocked down, supporting our hypothesis that DDX24 negatively regulates IRF7 dependent signaling which happens at a late stage of RLR signaling. Interestingly, we also observed an inhibitory role of DDX24 at the early time point, which is possibly caused by DDX24-mediated inhibition of NF-κB. Furthermore, we observed a decrease in DDX24 knockdown efficiency, especially in the 9 hour-treated group, possibly due to the induction of DDX24 by the treatment, which could have attenuated the effects of DDX24 at later times following poly I:C treatment. Therefore, the generation of DDX24 deficient cells would be crucial for clarification of this observation and further analysis.

Previously, FADD has been shown to play key roles in multiple cytoplasmic signaling processes such as apoptosis and innate immunity. The possible role of DDX24 in alternate mechanisms is underscored by demonstrating that DDX24^−/−^ mice die early in embryonic development (<e7.5). Indeed, we were unable to obtain DDX24^−/−^ MEFs for analysis. The severe embryonic phenotype clearly indicates that DDX24 is crucial to early embryogenesis. However, RLR deficient animals remain viable again suggesting that DDX24 has alternate functions in the cell [9], [46]. It is noteworthy that FADD deficient mice also exhibit early embryonic lethality, but at later stage from E9.5 to E11.5 possibly due to a severe failure of cardiac development [47].It remains to be seen whether DDX24 plays a role in these processes. Finally, it is worth noting that the negative regulation of innate immune gene transcription may play an important role in inflammatory disease. For example, defects in cytosolic DNA signaling that facilitate enhanced STING activity can lead to lethal inflammatory disease [48]. Mutations in the genes that negatively regulate STING have been found in patients suffering from severe inflammatory disease [49]. It remains to be seen whether defects in the RLR pathway may exert similar effects.

In summary, we have further characterized a new IFN-inducible DExD/H helicase DDX24 that is involved in a negative-feedback role to regulate the RLR pathway and type I IFN production. Further understating these processes may shed light in causes of infectious disease and plausibly inflammatory disorders involving enhanced innate immune gene activity.

## Materials and Methods

### Ethics statement

The protocol was reviewed and approved by the University of Miami Institutional Animal Care and Use Committee (IACUC) (Protocol number: 11-043 RENEWAL 03). This study was carried out in strict accordance with the recommendations in the Guide for the Care and Use of Laboratory Animals of the National Institutes of Health.

### Yeast two-hybrid assay

Yeast two-hybrid assay was performed using the Matchmaker Gal4 two-hybrid system (Clontech) according to the manufacturer protocols. Briefly, full length hFADD was cloned into the yeast bait vector, pGBT9. pGBT9-FADD transformed yeasts were transfected with another plasmid pGAD-hDDX24 or pGADT7 vector. Yeasts with both plasmids transfected were selected on SD-Leu/-Trp plates. Single colonies on the plates were picked up and seeded on SD-His/-Leu/-Trp plates for verification of interactions between molecules.

### Generation of DDX24 deficient mice

Gene trap mutated DDX24 embryonic stem cells (RRK059) were purchased from BayGenomics. Chimera mice were produced by microinjection of heterozygous ES cells into E3.5 C57BL/6 blastocysts that were subsequently transferred to pseudo-pregnant foster mothers. Chimera male mice were bredwith control female C57BL/6 mice to transmit the mutated *ddx24* alleleto the germline. Heterozygous mice were interbred to obtain DDX24^−/−^ mice. Genotyping was performed by genomic DNA based PCR. The primer 5′-GCTAATTCCTGCCTGTATGACCTT-3′ was used in combination with either 5′-ATTCAGAGCAGGTTAACCCAGGAC-3′ for the wild type *ddx*24 allele, or the primer 5′-GACTGGTGAGTACTCAACCAAGTC-3′ for the mutant allele. Animals were generated at the University Of Miami School Of Medicine Transgenic Core Facility (Miami, FL). Mice were allowed to access food and water freely and were housed at an ambient temperature of 23°C and at a 12 hour light/dark cycle. Animal care and handling was performed as per Institutional Animal Care and Use Committee guidelines.

### Isolation of murine embryonic fibroblasts (MEFs)

Timed matings were performed between mature DDX24 heterozygote mice and MEFs were obtained using a standard procedure. Briefly, embryos from E8.5 to E12.5 days were dissected free of surrounding tissues, washed in PBS and the heads and livers were removed. Each individual embryo was completely trypsinized for 15 minutes and cultured separately. MEFs were genotyped using same gDNA based method as described above. Primary DDX24^+/+^ and DDX24^+/−^ MEFs before passage 6 were used for all experiments.

### Histology and immunohistochemistry

Uteri from timed mating were formalin fixed and embedded in paraffin. Sections were cut at 6 µm. After dewaxing in xylene and rehydration in a series of graded ethanol, intermittent sections were stained with hematoxylin and eosin (H&E) in order to identify mutant embryos. Immuno-staining was performed after a heat and citrate based antigen retrieval of sections. The primary antibody used to detect Disabled-2 (Dab2) was a mouse monoclonal antibody from BD Transduction Laboratories. The secondary antibody applied was a peroxidase conjugate (Vector Labs, CA) and sections were counterstained with hematoxylin.

### Isolation of ES cells

After timed mating of DDX24 heterozygotes, preimplantation embryos were flushed at E3.5. The embryos were cultured upon an irradiated fibroblast feeder layer in ES cell media (DMEM with 15% FBS, 1000units/ml ESGRO, 1× non-essential amino acids, 2 mM L-glutamine, 50 IU/ml penicillin and 50 mg/ml streptomycin) until they had attached. They were then trypsinized, routinely fed and assayed for the appearance of ES cell clones.

### Cell culture, reagents, and antibodies

293T cells (ATCC), WT MEFs, DDX24^+/+^ and DDX24^+/−^ MEFs were grown in DMEM supplemented with fetal bovine serum (10%) and penicillin/streptomycin (1%). HUVEC cells were purchased from ATCC and cultured in EGM-2 media supplied with growth factors obtained from EGM Bullet kit. All cells were maintained at 37°C in a 5% CO_2_ laboratory incubator subject to routine cleaning and decontamination. Poly I:C (Amersham) was reconstituted in PBS at 2 mg/ml, denatured at 55°C for 30 min, and allowed to anneal at room temperature before use. Antibodies were obtained from following sources: rabbit anti-DDX24 (A300-697A Bethyl); mouse anti-FLAG M2 antibody, rabbit anti-c-Myc, mouse anti-HA (Sigma);rabbit anti-IRF3, rabbit anti-GFP, mouse anti-GAPDH (Santa Cruz Biotechnology, Inc.); rabbit anti-phospho-IRF3 (Upstate); rabbit anti-phospho-IRF7(Cell signaling); rabbit anti-Fibrillarin (ab5821), mouse anti β-actin (Abcam); mouse anti-RIP1 (BD Science); mouse anti-FADD(cell signaling). Control scrambled (D-001206–01-80), mRIG-I (L-065328-01), mDDX24 (L-042299-01) and hDDX24 (L-010397-01) smart pool siRNAs were purchased from Dharmacon/Thermo Scientific.

### Transfections and virus infections

Plasmid or poly I:C transfection in 293T cells or MEFs were conducted using Lipofectamine 2000 (Invitrogen) transfection reagents in Opti-MEM (Invitrogen) according to the manufacturer's manual. HUVEC and MEFs siRNA transfections were performed using AMAXA HUVEC and MEF Nucleofectin Kit 1 according to the manufacturer's recommendations (AMAXA Biosystems). Indiana strain of VSV was used in all experiments. Constructed VSVs (VSV-GFP, VSV-luc and VSVdM) were constructed in our lab. Briefly, keep the medium serum free during first two hours post infection and change the medium back to full medium until harvest.

### Plasmid constructs

Expression vectors (pcDNA3.1, Invitrogen) FLAG or GFP tagged RIG-I, MDA-5, dRIG-I, dMDA5, IPS1, TBK1, RIP1, FADD, IRF3 were generated in our lab by polymerase chain reaction. N-terminal c-Myc-tagged or FLAG-tagged plasmids DDX24, DDX24-N (AA 1–577) and DDX24-C (578–859) were generated using pCMV-tag system from stratagene. Same fragment of DDX24 were also ligated into the EcoRI and SalI site of pGADT7 vector to generate pGADT7-DDX24 for yeast two hybrid experiments. Other plasmids used in this study, c-Myc-RIP1, c-Myc-IRF7, FLAG-IRF7, and IFNα4-luc (S Ning); IFN-β Luc (J Hiscott). IRF3 (SA) was purchased from invivogen.

### Real-time PCR

Total RNA was isolated by using RNeasy RNA extraction kit (Qiagen) and cDNA synthesis was performed with random hexamer primers using 5 µg of total RNA (Invitorgen) Real-time PCR was performed using a LightCycler 2.0 instrument and the TaqMan Gene Expression Assays (Applied Biosystems): mIFNβ (Mm00439546), mIFNα2 (Mm00833961), mIRF7 (Mm00516788), mRIG-I (Mm00554529), mDDX24 (Mm00517454), hIFNhβ (Hs00185375), hIRF7 (Hs00185375), Luciferase (4331348 customized). Relative amount of mRNA was normalized to the 18S ribosomal RNA level in each sample. Alternatively, SYBR green systems from New England Biolabs (DyNAmo SYBR Green qPCR Kit) were used for human *ddx24* detection. The human RNA samples used for *ddx24* profiling were purchased from Ambion (AM6000FirstChoice Human Total RNA Survey Panel). Primers used for human ddx24 were: Forward 5′-GCCGAATTTACAGGAATTAAAACTG-3′; Reverse 5′-GTCATCCACTACCAGGGCACCTGAGC-3′. Primers used for human *gapdh*were Forward 5′-atgacatcaagaaggtggtg-3′; Reverse 5′-cataccaggaaatgagcttg-3′. Relative amount of mRNA was normalized to the *gapdh* RNA level in each sample.

### Reproter assays and immunofluorescence

Briefly, 293T cells or MEF cells were seeded on 24-well plates and were transiently transfected with 50 ng/100 ng of the luciferase reporter plasmids together with a total of 600 ng of various expression plasmids or empty control plasmids. As an internal control, 10 ng/20 ng pRL-TK plasmids expressing Renilla protein was transfected simultaneously. Twenty four or 36 hours later, cells were lysed by adding 100 µl/well of Cell culture lysis buffer (CCLR), and luciferase activity in the total cell lysate was measured by illuminometer.

Immunofluorescence experiments were performed as follows. Briefly, cell monolayers were fixed in 4% paraformaldehyde for 10 min, washed with PBS and permeabilized with 0.2% Triton-X 100 in PBS for 5 min. After blocking in PBS containing 10% FBS for 20 min, samples were incubated 1 hour at 37°C or overnight at 4°C with appropriate primary antibody. After PBS washing for three times, samples were incubated 1 hour with secondary antibodies conjugated with Cy3, Cy5 or FITC at 1: 200 dilution. Cells were washed again and incubated with 0.5 µg/ml DAPI solution for 5 min. Samples were then washed with PBS and mounted using prolong gold antifade reagent from invitrogen. Pictures were taken using an Olympus fluorescent microscope equipped with a digital camera and a Zeiss LSM-510 Confocal Laser Scanning Microscope.

### Immunoblotting, co-immunoprecipitations and RNA pulldown assay

Cells were lysed in RIPA buffer on ice, followed by centrifugation. Cell lysates were separated by SDS-PAGE, transferred to PVDF membranes, and subjected to immunoblotting. For co-immunoprecipitation, expression vectors were transfected into 293T cells for 36 to 48 hours, cells were lysed in ice-cold NP40 IP buffer (50 mM Tris, pH 8.0, 150 mM NaCl, 0.5% NP-40, 50 mM NaF, 0.1 mM Na_3_VO_4_, 1 mM DTT) or RIPA buffer with protease inhibitors (100 mM PMSF, Leupeptin, Aprotinine, Pepstatin), and cell lysates were precipitated with appropriate amount of FLAG-M2 antibody (SIGMA) or endogenous antibodies overnight at 4°C. Following day, 30 µl of Protein G was added and incubated for 3 hours. RNA pulldown assay was performed using the same lysis buffer and method. Cell lysates were incubated precipitated with biotin labeled RNA/DNA for 3 hours before precipitated with streptavidin beads. All precipitates were washed with lysis buffer 3 times and proteins were released by 2× Sample Buffer after boiling and analyzed by SDS-PAGE.

### Native PAGE gel dimerization assay

293T cells recovered from 6-well dishes were lysed in 100 µl of native lysis buffer (50 mM Tris-Cl, pH 8.0, 1% NP40, 150 mM NaCl, 100 mg/ml leupeptin, 1 mM PMSF, 5 mM orthovanadate). Ten µg of protein was mixed with 2× native PAGE sample buffer (125 mM Tris-Cl, pH 6.8, 30% glycerol, bromphenol blue) and subjected to electrophoresis on non-denaturing 7.5% polyacrylamide gels.

### ELISA

ELISAs for mouse IFNβ were performed using supernatants from cells where values are expressed as pg/ml ±S.E. as calculated from a standard curve derived from recombinant IFNβ provided in the ELISA kit (PBL Interferon Source).

### GENE array analysis and promoter analysis

Total RNA were purified and transcripts analyzed by Illumina Sentrix Chip Array (Mouse WG6 version2). Promoter analysis is performed by Genomatix MutInspector software.

### Statistical analysis

Statistical significance of differences in cytokine levels, mRNA levels, viral titers, and luciferase intensity in reporter assay and VSV-Luc-infected cells were determined using Student's t-test.

### Accession numbers

The following information was arranged in the format of “Symbol”, “Accession numbers (Human, Mouse)”. RIG-I (O95786, Q6Q899); MDA5(Q9BYX4, Q8R5F7); IPS-1/MAVS/CARDIF/VISA(Q7Z434, Q8VCF0); TBK-1(Q9UHD2, Q9WUN2); IKKi(Q14164, Q9R0T8); RIP1(Q13546, Q60855); FADD(Q13158, Q61160); DDX24(Q9GZR7, Q9ESV0); TRADD(Q15628, Q3U0V2); IRF3(Q14653, P70671); IRF7(Q92985, P70434); TLR3(O15455, Q99MB1); TLR7(Q9NYK1, P58681); TLR8(Q9NR97, P58682); LGP2(Q96C10, Q99J87); TLR9(Q9NR96, Q9EQU3); STING/TMEM173/MITA/MPYS(Q86WV6, Q3TBT3); CXCL10(P02778, P17515); IFNβ(P01574, P01575); DHX9(Q08211, O70133); DDX60(Q8IY21, E9PZQ1); DDX3X(O00571, Q62167); TRIF(Q8IUC6, Q80UF7); DHX36(Q9H2U1, Q8VHK9); MyD88(Q99836, P22366); DDX41(Q9UJV9, Q91VN6).

## Supporting Information

Figure S1 **Homology and expression profile of DDX24.** (A) Homology of human DDX24 and mouse DDX24 protein. (B) RNA expression profiling of ddx24 in different human organs by RT-PCR assays.
(PDF)

Figure S2 **DDX24 locates at both nuclear and cytoplasm.** (A) Immunofluorescence of DDX24 and FADD in MEFs. (B) Immunofluorescence of DDX24 and FADD under poly I:C treatment at different time points in MEFs. (C) Fractionation experiments in HUVEC cells indicate a nuclear and cytoplasmic localization of both DDX24 and FADD. (D) Endogenous IP of DDX24 and FADD with or without 6 hours poly I:C treatment. (E) Inductions of DDX24 by poly I:C and IFNβ are STAT1 dependent in MEFs. (F) Endogenous IP of DDX24 and TRADD in 293T cells.
(PDF)

Figure S3 **DDX24 binds to RNA.** The mixture of c-Myc-DDX24 and 1 mg/ml biotin-poly I:C were incubated without poly I:C or with polyI:C at 1 and 2 mg/ml concentration. Bound proteins were analyzed by immunoblotting with anti-c-Myc.
(PDF)

Figure S4 **DDX24 does not generally inhibits firefly luciferase activity or cell death.** (A) DDX24 inhibited poly I:C induced IFNβ promoter driven luc mRNA level by RT-PCR assay. (B) DDX24 does not inhibit Gal3 driven luc mRNA level by RT-PCR assay. (C) Overexpression of DDX24 does not affect percentage of live cells in 293T cells. (D) Overexpression of DDX24 does not affect cell growth in 293T cells.
(PDF)

Figure S5 **DDX24 blocks NF-κB signaling, but not IRF3 signaling.** (A) DDX24 inhibited poly I:C/VSVdM induced NF-κB-luc in 293T cells. (B) DDX24 does not inhibit IRF3(SA) triggered IFNβ-luc in 293T cells. (C) Expression of IRF3(SA) could reverse DDX24's inhibition of poly I:C triggered IFNβ-luc in 293T cells.
(PDF)

Figure S6 **Generation of DDX24-deficient mice by gene trapping.** (A) Genomic organization of the DDX24 locus. PCR primers for genotyping are indicated by arrows. (B) Genomic DNA-based PCR genotyping strategy for mice using primers described in materials and methods. (C) Genotyping of mouse embryos at E8.5. (D) Genotyping of mouse embryos at E3.5. (E) DDX24 deficient embryos exhibit abnormal develop at E7.5.
(PDF)

Figure S7 **Model depicting the proposed role of DDX24 in the inhibition of RLR signaling.** In response to viral infection, the RLR recognize viral nucleic acid and trigger a downstream signaling cascade, including the adaptor protein FADD and RIP1. DDX24 is recruited to FADD and RIP1 to form a regulatory complex, and attenuates RLR dependent signaling by either competing RNA ligand binding to RIG-I or impeding IRF7 activity through disrupting RIP1/IRF7 interactions. Additionally, DDX24 is upregulated by IFNβ, which suggests a negative feedback role in regulating RLR signaling.
(PDF)
