# Supplementary material for: DDX24 Negatively Regulates Cytosolic RNA-Mediated Innate Immune Signaling
Source: PLoS Pathog. 2013 Oct 31;9(10):e1003721. doi: 10.1371/journal.ppat.1003721 (PMC3814876; doi:10.1371/journal.ppat.1003721)
Supplement: Figure S1 — Homology and expression profile of DDX24. (A) Homology of human DDX24 and mouse DDX24 protein. (B) RNA expression profiling of ddx24 in different human organs by RT-PCR assays. (PDF) [file ppat.1003721.s001.pdf]

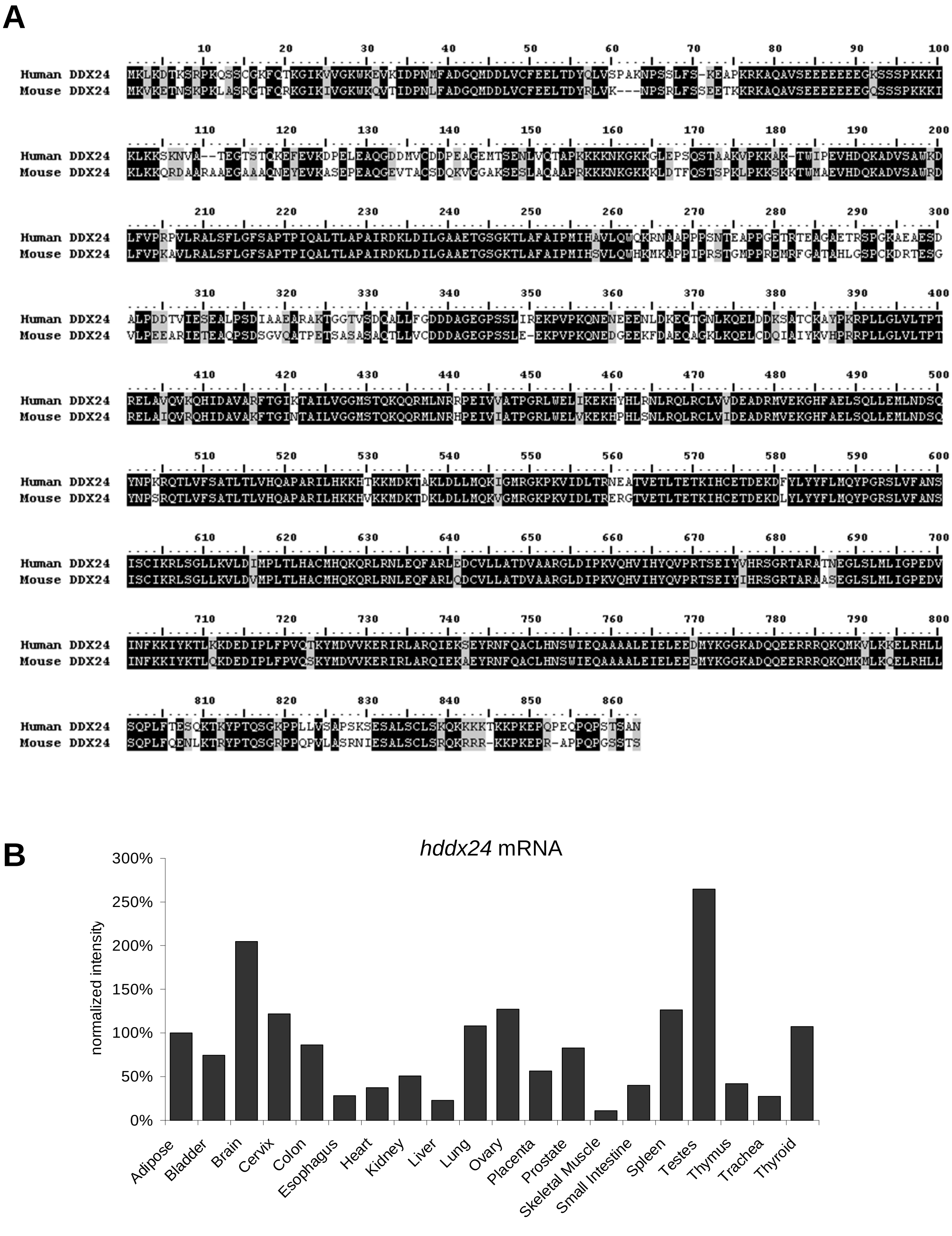
A
Human DDX24
Mouse DDX24
B
hddx24 mRNA
normalized intensity
300%
250%
200%
150%
100%
50%
0%
Adipose
Bladder
Brain
Cervix
Colon
Esophagus
Heart
Kidney
Liver
Lung
Ovary
Placenta
Prostate
Skeletal Muscle
Small Intestine
Spleen
Testes
Thymus
Trachea
Thyroid
