# Supplementary figures and images for: DDX24 Negatively Regulates Cytosolic RNA-Mediated Innate Immune Signaling
Source: PLoS Pathog. 2013 Oct 31;9(10):e1003721. doi: 10.1371/journal.ppat.1003721 (PMC3814876; doi:10.1371/journal.ppat.1003721)

**A**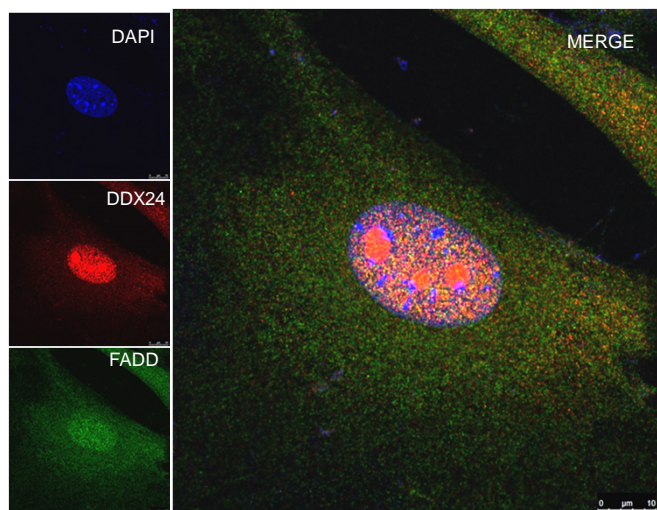

MEF

**B**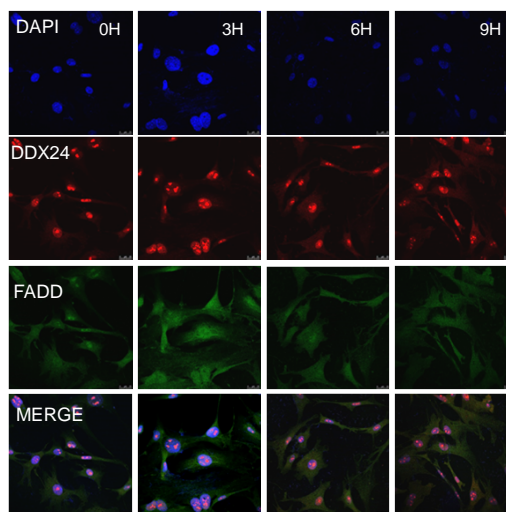

MEF poly I:C treatment

**C**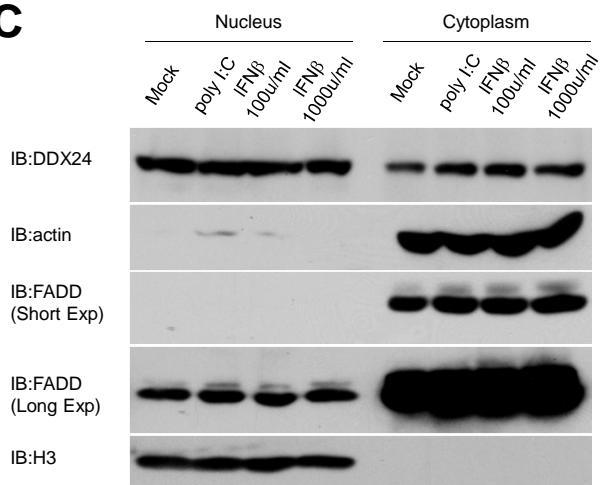

HUVEC

**D**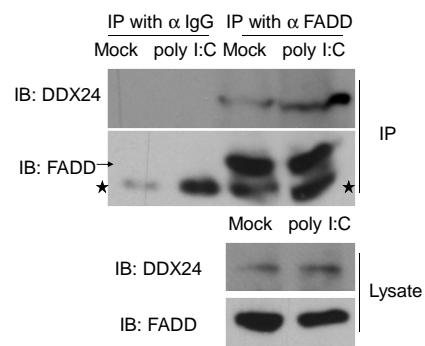

MEF

**E**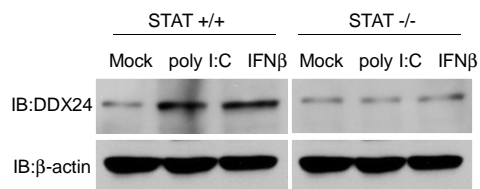

MEF

**F**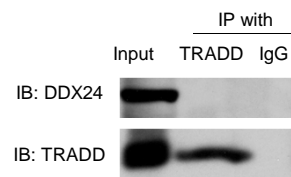

293T

Supplement: Figure S2 — DDX24 locates at both nuclear and cytoplasm. (A) Immunofluorescence of DDX24 and FADD in MEFs. (B) Immunofluorescence of DDX24 and FADD under poly I:C treatment at different time points in MEFs. (C) Fractionation experiments in HUVEC cells indicate a nuclear and cytoplasmic localization of both DDX24 and FADD. (D) Endogenous IP of DDX24 and FADD with or without 6 hours poly I:C treatment. (E) Inductions of DDX24 by poly I:C and IFNβ are STAT1 dependent in MEFs. (F) Endogenous IP of DDX24 and TRADD in 293T cells. (PDF) [file ppat.1003721.s002.pdf]

**A**

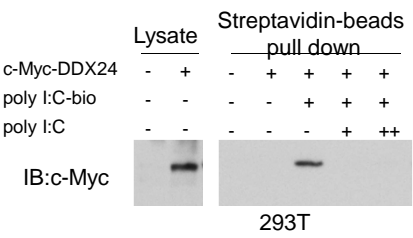

**B**

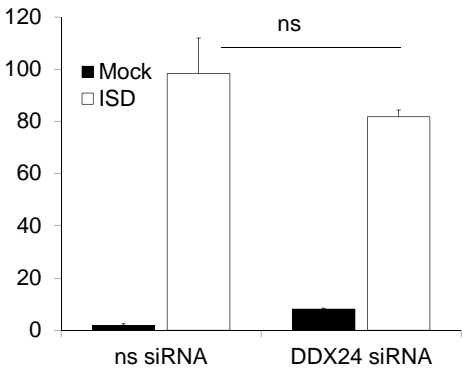

Supplement: Figure S3 — DDX24 binds to RNA. The mixture of c-Myc-DDX24 and 1 mg/ml biotin-poly I:C were incubated without poly I:C or with polyI:C at 1 and 2 mg/ml concentration. Bound proteins were analyzed by immunoblotting with anti-c-Myc. (PDF) [file ppat.1003721.s003.pdf]

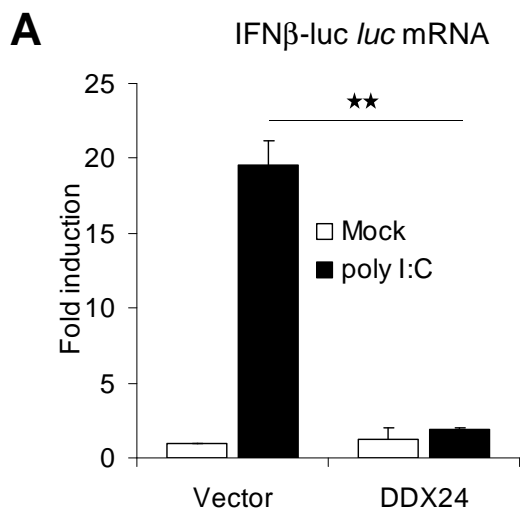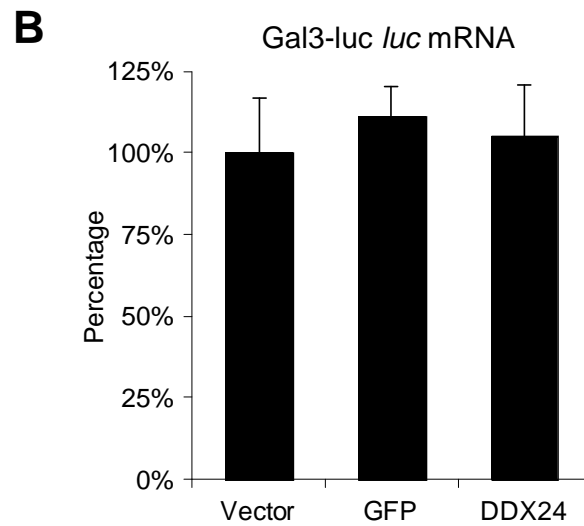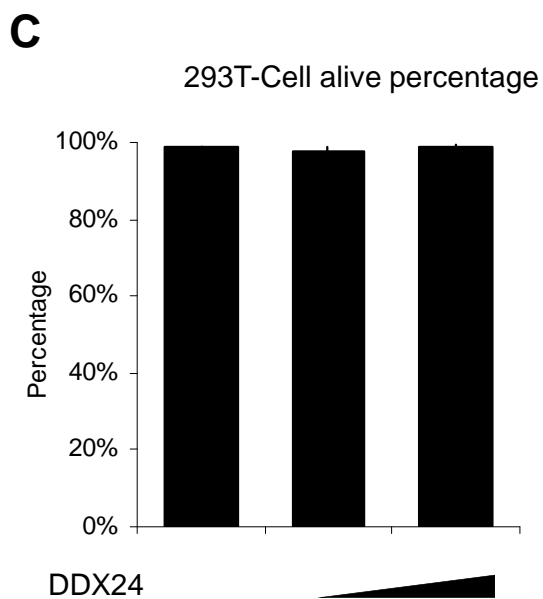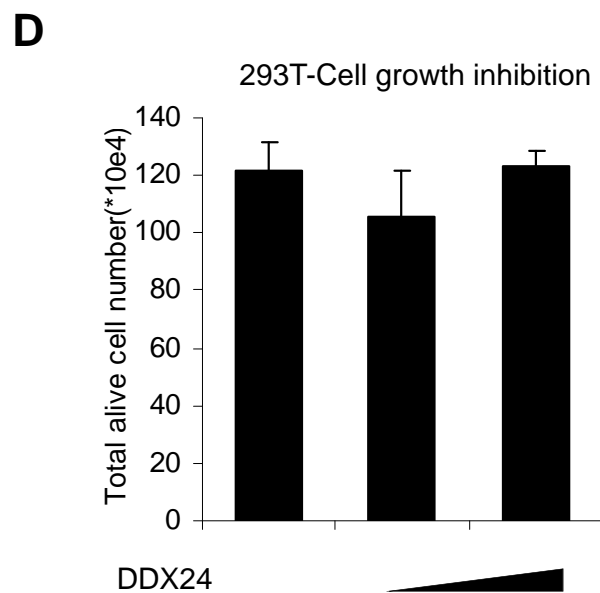

Supplement: Figure S4 — DDX24 does not generally inhibits firefly luciferase activity or cell death. (A) DDX24 inhibited poly I:C induced IFNβ promoter driven luc mRNA level by RT-PCR assay. (B) DDX24 does not inhibit Gal3 driven luc mRNA level by RT-PCR assay. (C) Overexpression of DDX24 does not affect percentage of live cells in 293T cells. (D) Overexpression of DDX24 does not affect cell growth in 293T cells. (PDF) [file ppat.1003721.s004.pdf]

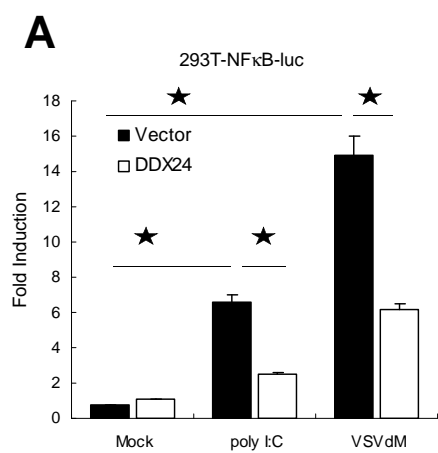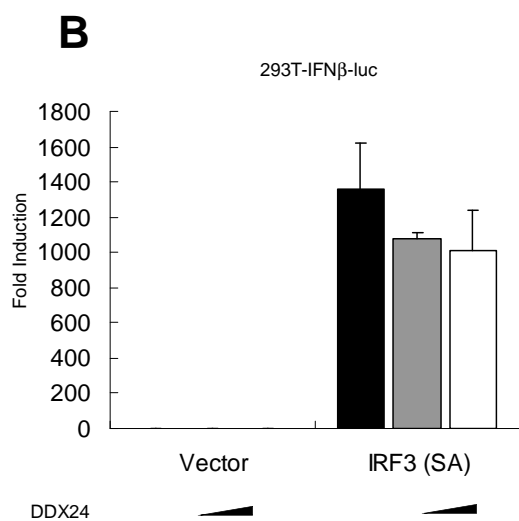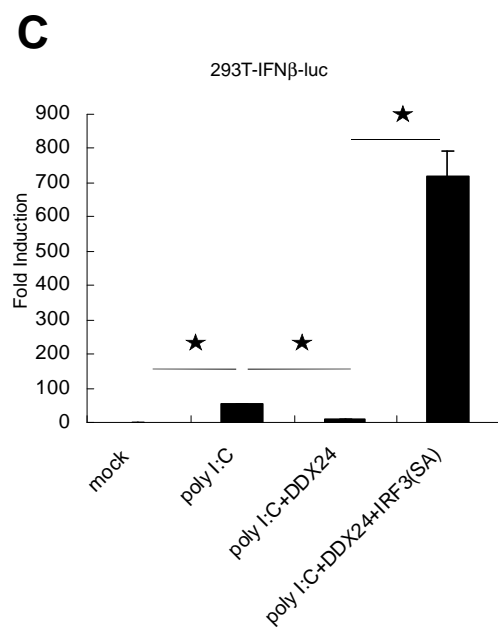

Supplement: Figure S5 — DDX24 blocks NF-κB signaling, but not IRF3 signaling. (A) DDX24 inhibited poly I:C/VSVdM induced NF-κB-luc in 293T cells. (B) DDX24 does not inhibit IRF3(SA) triggered IFNβ-luc in 293T cells. (C) Expression of IRF3(SA) could reverse DDX24's inhibition of poly I:C triggered IFNβ-luc in 293T cells. (PDF) [file ppat.1003721.s005.pdf]

**A**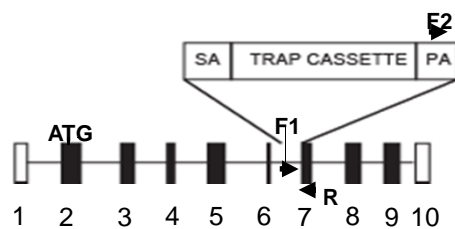**B**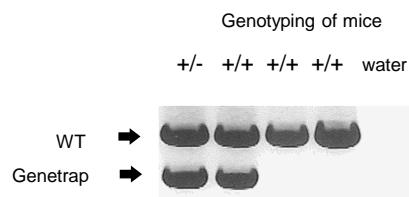**C**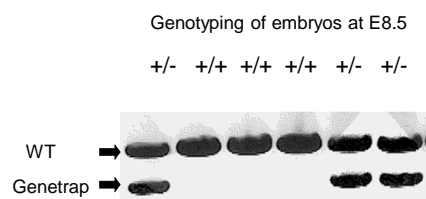**D**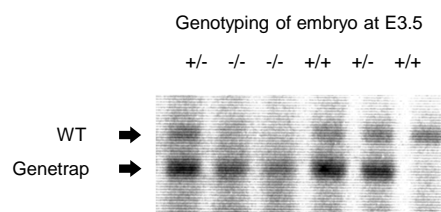**E**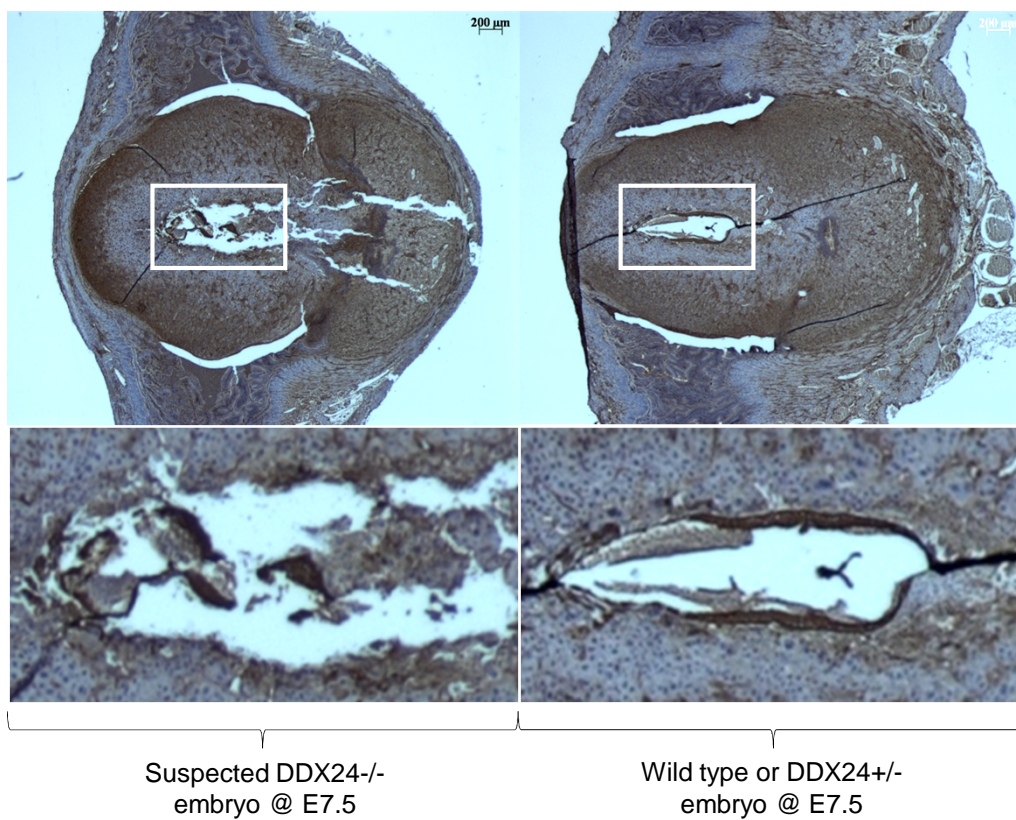

Supplement: Figure S6 — Generation of DDX24-deficient mice by gene trapping. (A) Genomic organization of the DDX24 locus. PCR primers for genotyping are indicated by arrows. (B) Genomic DNA-based PCR genotyping strategy for mice using primers described in materials and methods. (C) Genotyping of mouse embryos at E8.5. (D) Genotyping of mouse embryos at E3.5. (E) DDX24 deficient embryos exhibit abnormal develop at E7.5. (PDF) [file ppat.1003721.s006.pdf]

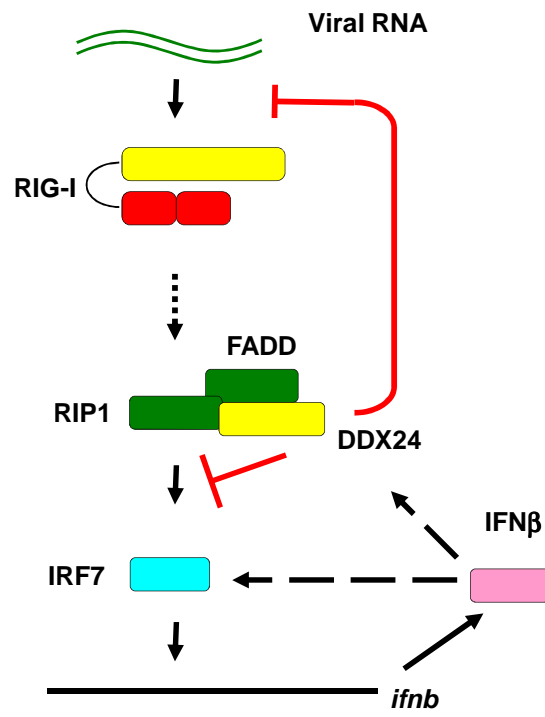

Supplement: Figure S7 — Model depicting the proposed role of DDX24 in the inhibition of RLR signaling. In response to viral infection, the RLR recognize viral nucleic acid and trigger a downstream signaling cascade, including the adaptor protein FADD and RIP1. DDX24 is recruited to FADD and RIP1 to form a regulatory complex, and attenuates RLR dependent signaling by either competing RNA ligand binding to RIG-I or impeding IRF7 activity through disrupting RIP1/IRF7 interactions. Additionally, DDX24 is upregulated by IFNβ, which suggests a negative feedback role in regulating RLR signaling. (PDF) [file ppat.1003721.s007.pdf]
